# Immune-related gene signature for predicting the prognosis of head and neck squamous cell carcinoma

**DOI:** 10.1186/s12935-020-1104-7

**Published:** 2020-01-17

**Authors:** Yangyang She, Xiangbo Kong, Yaping Ge, Ping Yin, Zhiyong Liu, Jieyu Chen, Feng Gao, Silian Fang

**Affiliations:** 1 Department of Oral and Maxillofacial Surgery, The Sixth Affiliated Hospital of Sun Yat-sen University, No. 26 Yuancun Erheng Rd, Guangzhou, 510655 Guangdong China; 2 Department of Stomatology, Sun Yat‑sen Memorial Hospital of Sun Yat-sen University, Guangzhou, Guangdong China; 3 Department of Colorectal Surgery, The Sixth Affiliated Hospital of Sun Yat-sen University, No. 26 Yuancun Erheng Rd, Guangzhou, 510655 Guangdong China; 4 Guangdong Provincial Key Laboratory of Colorectal and Pelvic Floor Diseases, The Sixth Affiliated Hospital of Sun Yat-sen University, No. 26 Yuancun Erheng Rd, Guangzhou, 510655 Guangdong China; 5 Section of Oral and Maxillofacial Surgery, Department of Surgery, University of Michigan, Ann Arbor, MI 48109 USA

**Keywords:** Immune related gene signature (IRGS), Prognosis, Head and neck squamous cell carcinoma (HNSCC)

## Abstract

**Background:**

Immune-related genes (IRGs) were linked to the prognosis of head and neck squamous cell carcinoma (HNSCC). This study aimed to identify the effects of an immune-related gene signature (IRGS) that can predict the of HNSCC prognosis.

**Methods:**

The expression data of 770 HNSCC patients from the TCGA database and the GEO database were used. To explore a predictive model, the Cox proportional hazards model was applied. The Kaplan–Meier survival analysis, as well as univariate and multivariate analyses were performed to evaluate the independent predictive value of IRGS. To explore biological functions of IRGS, enrichment analyses and pathway annotation for differentially expressed genes (DEGs) in different immune groups were applied, as well as the immune infiltration.

**Results:**

A prognostic signature comprising 27 IRGs was generated. IRGS significantly stratified HNSCC patients into high and low immune risk groups in regard to overall survival in the training cohort (HR = 3.69, 95% *CI* 2.73–4.98, *P *< 0.001). Likewise, IRGS could be linked to the prognosis of HNSCC in patients of the validation cohort (HR = 1.84, 95% *CI* 1.21–2.81, *P *< 0.01). Even after adjusting for TNM stage, IRGS was maintained as an independent predictor in the multivariate analysis (HR = 3.62, 95% *CI* 2.58–5.09, *P *< 0.001), and in the validation cohort (HR = 1.73, 95% *CI* 1.12–2.67, *P *= 0.014). The IFN-α response, the IFN-γ response, IL-2/STAT5 signaling, and IL-6/JAK/STAT3 signaling were all negatively correlated with the immune risk (*P *< 0.01). Immune infiltration of the high-risk group was significantly lower than that of the low-risk group (*P *< 0.01). Most notably, the infiltration of CD8 T cells, memory-activated CD4 T cells, and regulatory T cells was strongly upregulated in the low immune risk groups, while memory resting CD4 T cell infiltration was downregulated (*P *< 0.01).

**Conclusion:**

Our analysis provides a comprehensive prognosis of the immune microenvironments and outcomes for different individuals. Further studies are needed to evaluate the clinical application of this signature.

## Background

Head and neck squamous cell carcinomas (HNSCCs) represent a group of malignancies in sites of the oral cavity as well as the nasopharynx, oropharynx, hypopharynx, and larynx. Worldwide, more than 600,000 patients are diagnosed with HNSCC every year. Thus, it ranks as the sixth most common form of cancer [1, 2]. Traditionally, the formation of HNSCC is linked to smoking and alcohol consumption. Recently, there is accumulating evidence suggesting that the human papillomavirus (HPV) presents a vital etiological factor in some patients [3]. The 5-year survival rate of HNSCC is approximately 60%, with 380,000 deaths annually [1, 4, 5]. A significant cause of mortality is loco-regional recurrence. For patients suffering recurrent metastatic diseases, the median overall survival (OS) is only 10 to 13 months in the first-line chemotherapy setting and 6 months in the setting of second-line [6]. Moreover, long-term toxicity and morbidity may be induced by the treatment [7]. As a consequence, exploring a novel and reliable signature for prognosis is critical.

Gene expression signatures for survival stratification in HNSCC patients have been proposed by various studies. Elements of the immune system, such as the tumor immune evading mechanism, are increasingly recognized crucial in HNSCC progression [7–9]. The programmed cell death protein 1/programmed cell death ligand 1 (PD-1/PDL-1) complex is part of an important immune-checkpoint which is involved in antitumor activity [10]. Importantly, the anti-PD-1 antibodies pembrolizumab and nivolumab were approved for treating platinum-based chemotherapy refractory recurrent or metastatic HNSCC by the US Food and Drug Administration (FDA) in 2016 [11, 12]. However, the objective response rates to checkpoint blockade immunotherapy only range from 16 to 25% [11, 12]. As recent studies indicated, immune-related biomarkers could define not only the patients’ immune state, but also the biological behavior of HNSCC [13–15]. For example, tumor-infiltrating lymphocytes (TILs) in the tumor microenvironment could contribute to improved prognosis [14]. However, the molecular characteristics depicting tumor immune interaction remain largely unknown, specifically in regard to the prognostic potential for HNSCC. Indeed, it is generally believed that an individual’s immune state is too complex to be illustrated by a single immune marker.

Therefore, immune-related genes from a rich supply of HNSCC transcriptional data were analyzed in this study. In order to construct a new signature to facilitate prognosis, combinatorial immune biomarkers were explored and developed. Furthermore, the prognostic prediction significance of this immune-associated gene marker system was systematically validated. This presents a critical step towards developing personalized strategies to improve therapeutic outcomes for HNSCC patients.

## Materials and methods

### Patients

The gene expression profiles of fresh frozen HNSCC tumor tissue samples from 2 public cohorts involving 770 HNSCC patients were retrospectively analyzed. The largest individual data set training, namely, the Cancer Genome Atlas HNSCC (TCGA HNSCC data set, n = 500), was selected for training. The remaining microarray data set (GSE65858, n = 270) was chosen to serve as a validation cohort. GSE65858 was obtained in its processed form from Gene Expression Omnibus (GEO) using Bioconductor package ‘GEOquery’. The level 3 RNA expression profile data of the TCGA HNSCC cohort was downloaded from Broad GDAC Firehose (http://gdac.broadinstitute.org/) and log2-transformed transcripts per million (TPM) were utilized. In all data sets, survival analyses were only performed for patients for whom survival information was accessible. Paper charts as well as electronic medical records was examined when necessary. Information on HPV status for the TCGA cohort were updated according to detection of viral transcripts in RNA sequencing data [16]. ‘Combat’ in R package ‘sva’ was used to remove batch effects. Data were collected from December 20, 2018 to March 20, 2019.

### Construction and validation of an individualized prognostic signature based on IRGs

A predictive immune-related signature was constructed by concentrating on immune-related genes (IRGs) obtained from the Immunology Database and Analysis Portal (ImmPort) (https://immport.niaid.nih.gov). The IRGs measured by all platforms included in this study were selected. Prognostic IRGs were further screened by performing 1000 randomizations (each with 80% of all patients) and analyzed by the Cox proportional hazards model to estimate the correlation between each IRG and patients’ OS in the training data set. Since molecular signatures may be shared across stages, HNSCC in all stages were included.

The potential prognostic IRGs with *P*-values < 0.05 were used as candidates for the construction of the IRGS. To minimize the over-fitting risk and build a risk model for patients in all stages, we combined the least absolute shrinkage and selection operator (LASSO) with the Cox proportional hazards regression model to analyze all stage HNSCC samples. A tenfold cross-validation was used to estimate the penalty parameter in the training data set at the minimum partial likelihood deviance.

### Validation of IRGS

To divide patients into low-risk and high-risk groups, the optimal IRGS cutoff was analyzed via a time-dependent receiver operating characteristic (ROC) curve at the 5-year timepoint in the training data set. The ROC curve was estimated through the Kaplan–Meier method. The cutoff value was defined as the IRGS corresponding to the minimum distance between the ROC curve and point standing for the 100% true positive rate and 0% false-positive rate.

The predictive value of the IRGS was evaluated by univariate analyses for HNSCC patients in all stages in the training and validation cohort. Subsequently, IRGS was combined with clinical and pathologic features in multivariate analyses.

### Functional annotation and analysis

To explore biological functions of the IRGS, enrichment analyses and pathway annotations for differentially expressed genes (DEGs) in different immune groups were applied using the R package ‘gProfileR’ for the TCGA HNSCC data-set. High- risk and low-risk immune groups were predicted by the Gene Set Enrichment Analysis (GSEA) by IRGS using the Bioconductor package ‘HTSanalyzeR’ [17]. We examined gene sets of cancer hallmarks from MSigDB [18]. According to the IRGS system, patients were divided into different groups based on the associated immune risk. In the TCCA HNSCC data set, RNA sequencing data as well as data on the infiltration percentages of specific immune cells, such as lymphocytes, monocytes and neutrophils, are available for HNSCC tumor samples. Estimation of Stromal and Immune cells in Malignant Tumor tissues using Expression data (ESTIMATE) [19] was applied to estimate the proportions of immune and stromal cells. CIBERSORT, an established algorithm, was used to perform immune cell type-specific analyses. One-tailed t tests were applied to compare these pathologic features of HNSCC patients within different immune risk groups.

### Statistical analysis

Statistical analyses were performed using R software (version 3.5.1; http://www.Rproject.org). We computed descriptive statistics for all variables. These comprised means and standard deviations (SD), or medians and interquartile ranges (IQR) for continuous factors, as well as frequencies for categorical factors. LASSO regression was enforced using the ‘glmnet’ R package (version 2.0-16). Log-rank tests were used to evaluate univariate analysis of the link between IRGS and clinical pathologic features with OS. The R package ‘survivalROC’ (version 1.0.3) was applied to perform a time-dependent ROC curve analysis. The multivariate analysis was implemented with the Cox proportional hazards regression model for characteristics that were significantly associated with OS in univariate analyses. A *P*-value of < 0.05 was identified as statistically significant.

## Results

### Construction and definition of the IRGS

**Fig. 1:**
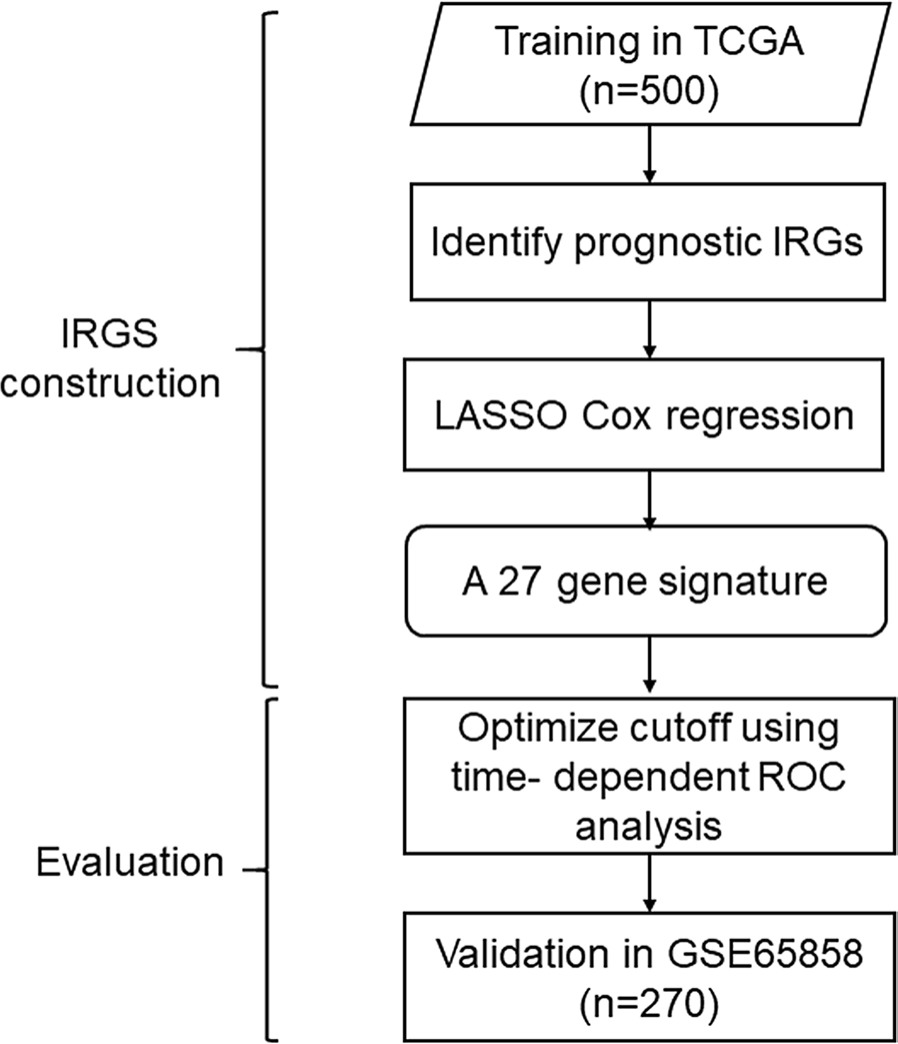
Establish and verification of IRGS. A schematic flow chart of study design and analysis steps

Based on the GEO dataset (GSE65858), a total of 270 eligible HNSCC patients in all stages were included in this study as part of the training cohort. Among 1073 immune genes measured on all platforms, 915 IRGs were selected after filtering median absolute deviation (MAD) > 0.5. By 1000 times resampling, 81 IRGs were robustly associated with individual patients’ OS. Employing LASSO Cox regression, 27 prognostic IRGs were chosen and combined to construct the IRGS (Fig. 1, Additional file 1: Figure S1). Subsequently, the prognosis correlation coefficient of each gene in IRGS was obtained (Table 1). With an optimal cutoff value of 0.106 to stratify low immune risk and high immune risk groups, prognostic significance was satisfactory at the 5-year timepoint, as suggested by the time-dependent ROC curve analysis (Additional file 2: Figure S2).

**Table 1:** 27-Gene immune signature

Gene	Name	Frequency in resampling	Average *P*-value	Coefficient
RFXAP	Regulatory factor X-associated protein	957	0.013	0.073
ULBP1	UL16 binding protein 1	927	0.016	0.007
TMSB4Y	Thymosin beta 4	1000	0.002	− 0.117
RBP4	Retinol binding protein 4	923	0.018	0.067
LCNL1	Lipocalin-like 1	1000	0.002	− 0.056
CCR6	Chemokine (C-C motif) receptor 6	976	0.008	− 0.009
KLRK1	Killer cell lectin-like receptor subfamily K	1000	0.000	− 0.093
PTX3	Pentraxin-related gene	999	0.002	0.037
MASP1	Mannan-binding lectin serine peptidase 1	1000	7.447	− 0.203
HRG	Histidine-rich glycoprotein	938	0.016	0.043
CCL22	Chemokine (C-C motif) ligand 22	992	0.005	− 0.061
OLR1	Oxidized low density lipoprotein (lectin-like) receptor 1	996	0.005	0.040
ROBO1	Roundabout	923	0.017	− 0.026
BTC	Betacellulin	902	0.020	0.146
CHGB	Chromogranin B	1000	0.001	0.113
DKK1	Dickkopf homolog 1	1000	9.203	0.088
HBEGF	Heparin-binding EGF-like growth factor	905	0.022	0.109
INHBB	Inhibin beta B	946	0.015	0.009
PDGFA	Platelet-derived growth factor alpha polypeptide	1000	0.001	0.037
AVPR2	Arginine vasopressin receptor 2	999	0.002	− 0.043
IL20RA	interleukin 20 receptor	952	0.012	− 0.067
RORB	RAR-related orphan receptor B	947	0.015	− 0.010
TNFRSF18	Tumor necrosis factor receptor superfamily member 18	934	0.017	− 0.071
TNFRSF25	Tumor necrosis factor receptor superfamily member 25	987	0.006	− 0.051
TNFRSF4	Tumor necrosis factor receptor superfamily member 4	1000	0.002	− 0.054
SH3BP2	SH3-domain binding protein 2	999	0.002	− 0.004
ICOS	Inducible T-cell co-stimulator	984	0.008	− 0.017

### Validation of the IRGS as a prognostic factor for HNSCC patients

Two HNSCC transcriptional datasets including prognostic data were selected to evaluate prognosis. The TCGA dataset (n = 500, Additional file 3: Table S1) was selected as the training dataset, the GEO dataset was used as the validation cohort (n = 270, Additional file 3: Table S1). There was no significant difference between the two cohorts in regard to their clinicopathologic characteristics (Table 2, Additional file 4: Table S2).

**Table 2:** Univariate and multivariate analyses of IRGS, clinical and pathologic factors of patients in training cohort

Characteristic	Univariate		Multivariate	
	HR (95% *CI*)	*P* value	HR (95% *CI*)	*P* value
IRGS	3.69 (2.73–4.98)	< 0.001	3.62 (2.58–5.09)	< 0.001
Age	1.02 (1.00–1.03)	0.01	1.02 (1.00–1.03)	< 0.01
Gender	0.71 (0.53-0.96)	0.03	0.90 (0.65–1.26)	0.55
TNM stage	1.38 (1.14–1.65)	< 0.001	1.24 (1.02–1.50)	0.03
Pathological grading	1.14 (0.93–1.39)	> 0.05	NA	NA
Smoking	NA	NA	NA	NA
Alcohol abuse	1.01 (0.74–1.37)	> 0.05	NA	NA
HPV	1.20 (0.88–1.63)	> 0.05	NA	NA

**Fig. 2:**
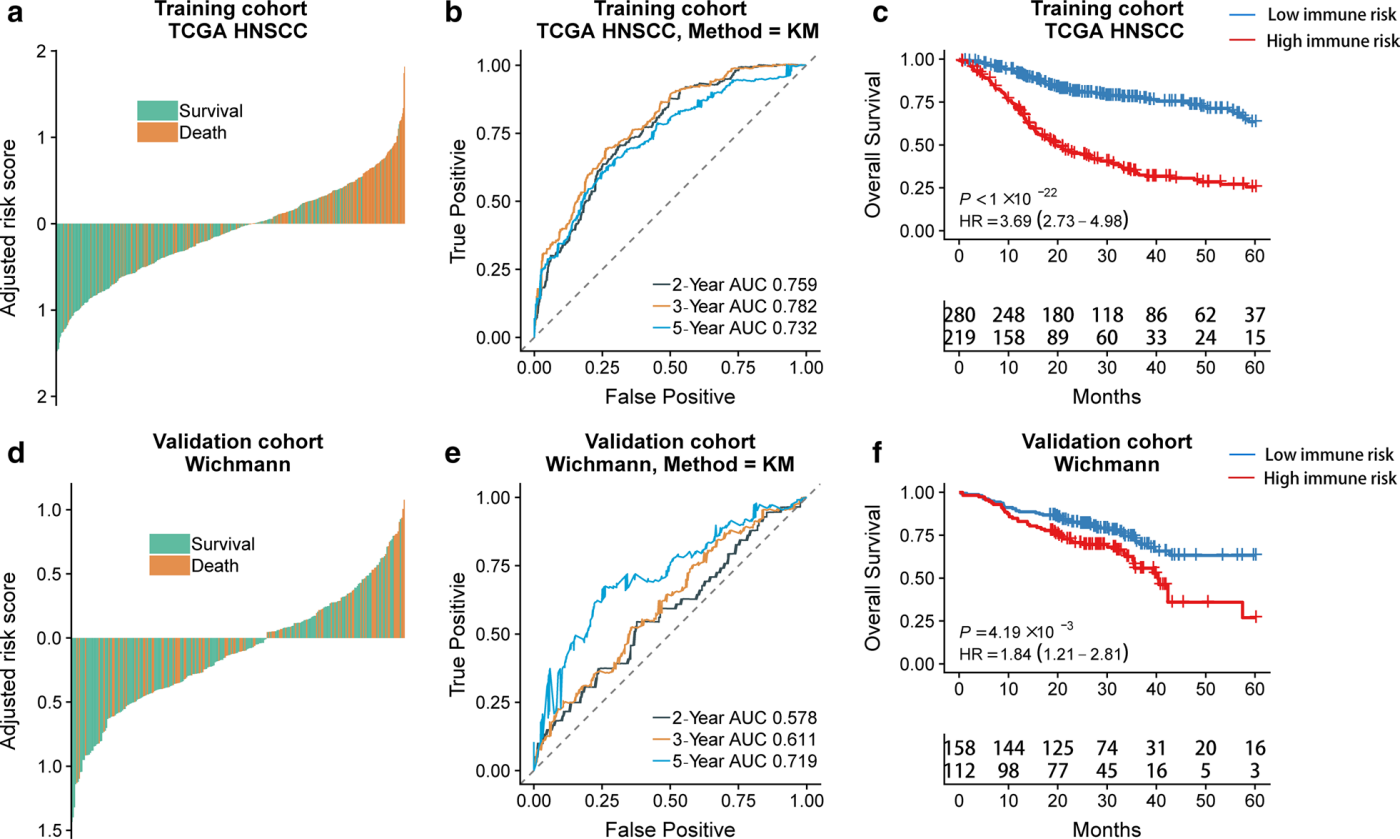
The outcomes of low and high immune risks in HNSCC patients. The overall survival rate of patients in the different immune risk groups of training cohort (**a**) and validation cohort (**d**). Kaplan–Meier curves comparing patients with low or high immune risk in training cohort (**b**) and validation cohort (**e**). IRGS significantly stratified HNSCC patients into low immune risk and high immune risk groups in regard to the overall survival in the training cohort (HR = 3.69, 95% *CI* 2.73–4.98, *P *< 0.001) (**c**), the validation cohort (HR = 1.84, 95% *CI* 1.21–2.81, *P *< 0.01) (**f**). *P*-values were calculated using log-rank tests and HR is short for hazard ratio

Among the HNSCC patients of training and validation cohorts, individuals of the immune high-risk group showed significantly higher adjusted risk scores for death than those in the immune low-risk group stratified by IRGS (Fig. 2a, d). In regards to 2-year, 3-year and 5-year follow-ups, a high prognostic value was also observed base on the time-dependent ROC curve method applied for the training cohort (AUC = 0.759 at 2 years; AUC = 0.782 at 3 years; AUC = 0.732 at 5 years) and validation cohort (AUC = 0.578 at 2 years; AUC = 0.611 at 3 years; AUC = 0.719 at 5 years) (Fig. 2b, e). IRGS significantly stratified HNSCC patients into low immune risk and high immune risk groups with respect to OS in the training cohort (HR = 3.69, 95% *CI* 2.73–4.98, *P *< 0.001), and in the validation cohort (HR = 1.84, 95% *CI* 1.21–2.81, *P *< 0.01) (Fig. 2c, f).

### IRGS as an independent risk factor for HNSCC patients

As we expected, IRGS, age and tumor stage were associated with the outcomes for HNSCC patients. In the univariate analysis, IRGS were related to OS in the training cohort (HR = 3.69, 95% *CI* 2.73–4.98, *P *< 0.001, Table 2). Similarly, it was found that IRGS was linked to OS in the validation cohort (HR = 1.84, 95% *CI* 1.21–2.81, *P *< 0.01, Additional file 4: Table S2). Despite adjustment for the TNM stage in the multivariate analysis, IRGS was maintained as an independent predictor in the training cohort (HR = 3.62, 95% *CI* 2.58–5.09, *P *< 0.001, Table 2) and in the validation cohort (HR = 1.73, 95% *CI* 1.12–2.67, *P *= 0.014, Additional file 4: Table S2).

### HPV as a risk factor for HNSCC patients

In the univariate analysis, HPV was not significantly associated with the prognosis for the training cohort (HR = 1.20, 95% *CI* 0.88–1.63, *P *> 0.05, Table 2). It was, however, associated with better survival outcome in the validation cohort (HR = 1.95, 95% *CI* 1.15–3.33, *P *< 0.05, Additional file 4: Table S2). In the same cohort, when incorporated with other clinicopathologic features, it showed to be significantly linked to the prognosis in multivariate analysis (HR = 2.15, 95% *CI* 1.24–3.72, *P *< 0.01, Additional file 4: Table S2).

### Functional annotation of the IRGS

**Fig. 3:**
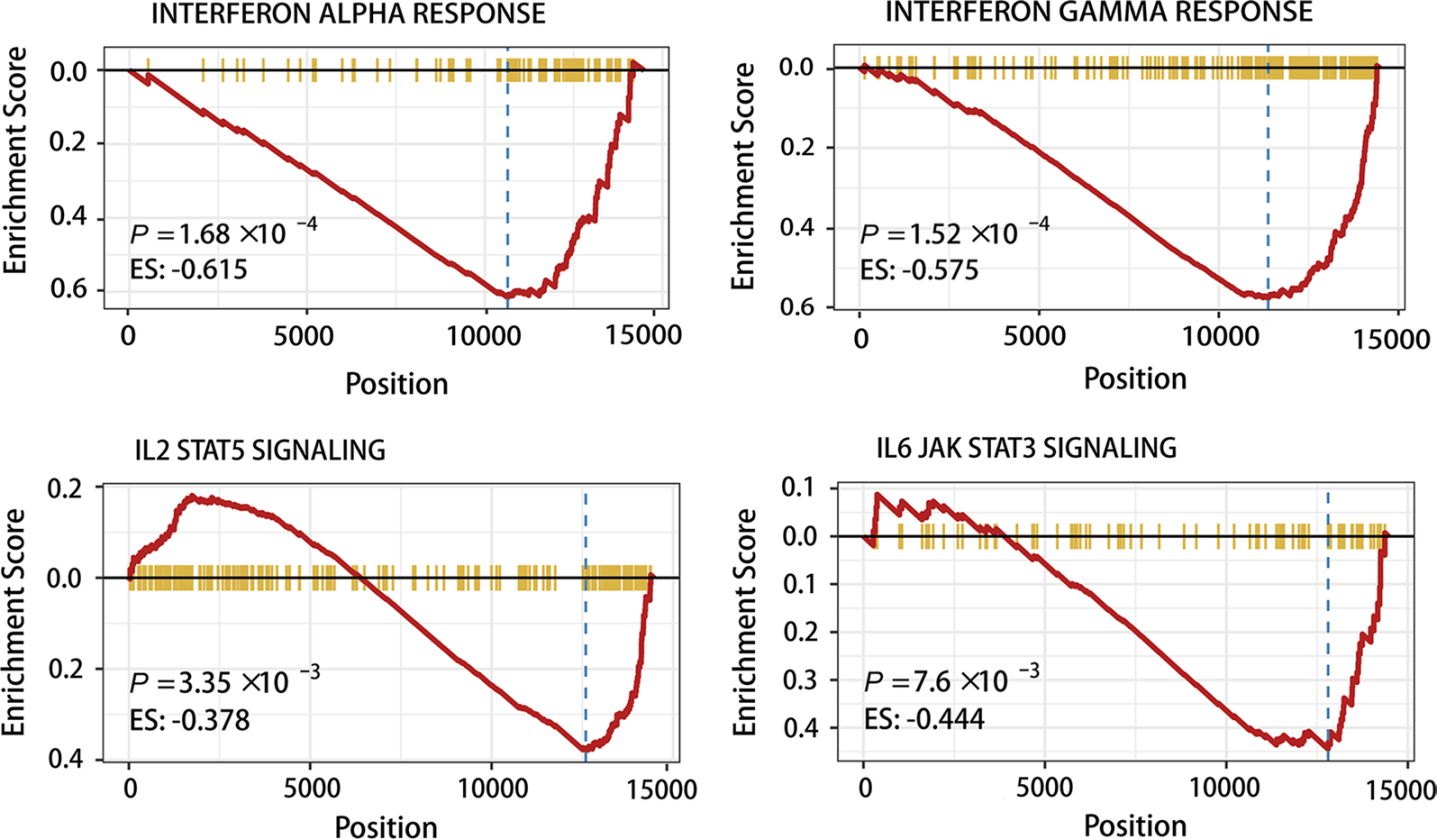
Functional annotation of the IRGS. GSEA analysis showed the IFN-α response, the IFN-γ response, IL-2 STATS signaling and IL-6 JAK STAT3 signaling were depressed in high immune risk patients. ES is short for enrichment score

27 IRGs were included in the IRGS, including UL16-binding protein 1 (ULBP1), chemokine receptors 6 (CCR6), C-C motif chemokine ligand 22 (CCL22), roundabout guidance receptor 1 (ROBO1), dickkopf WNT signaling pathway inhibitor 1 (DKK1) and platelet derived growth factor subunit A (PDGFA), all of which have previously been shown to be correlated to the pathogenesis and progression of HNSCC (Table 1). Moreover, GSEA has been implicated in multiple biological processes that show either a positive or negative correlation with the immune risk in hallmarks of HNSCC. The most beneficial biological functions, condition and signaling pathways included hypoxia, the interferon alpha (IFN-α) response, the interferon-γ (IFN-γ) response, IL-2/STAT5 signaling, IL-6/JAK/STAT3 signaling, epithelial mesenchymal transition, TGF-β signaling, and hedgehog signaling (Fig. 3, Additional file 5: Table S3). Interestingly, IFN-α, IFN-γ, IL-2 and IL-6 were downregulated in patients with a high immune risk (Fig. 3).

**Fig. 4:**
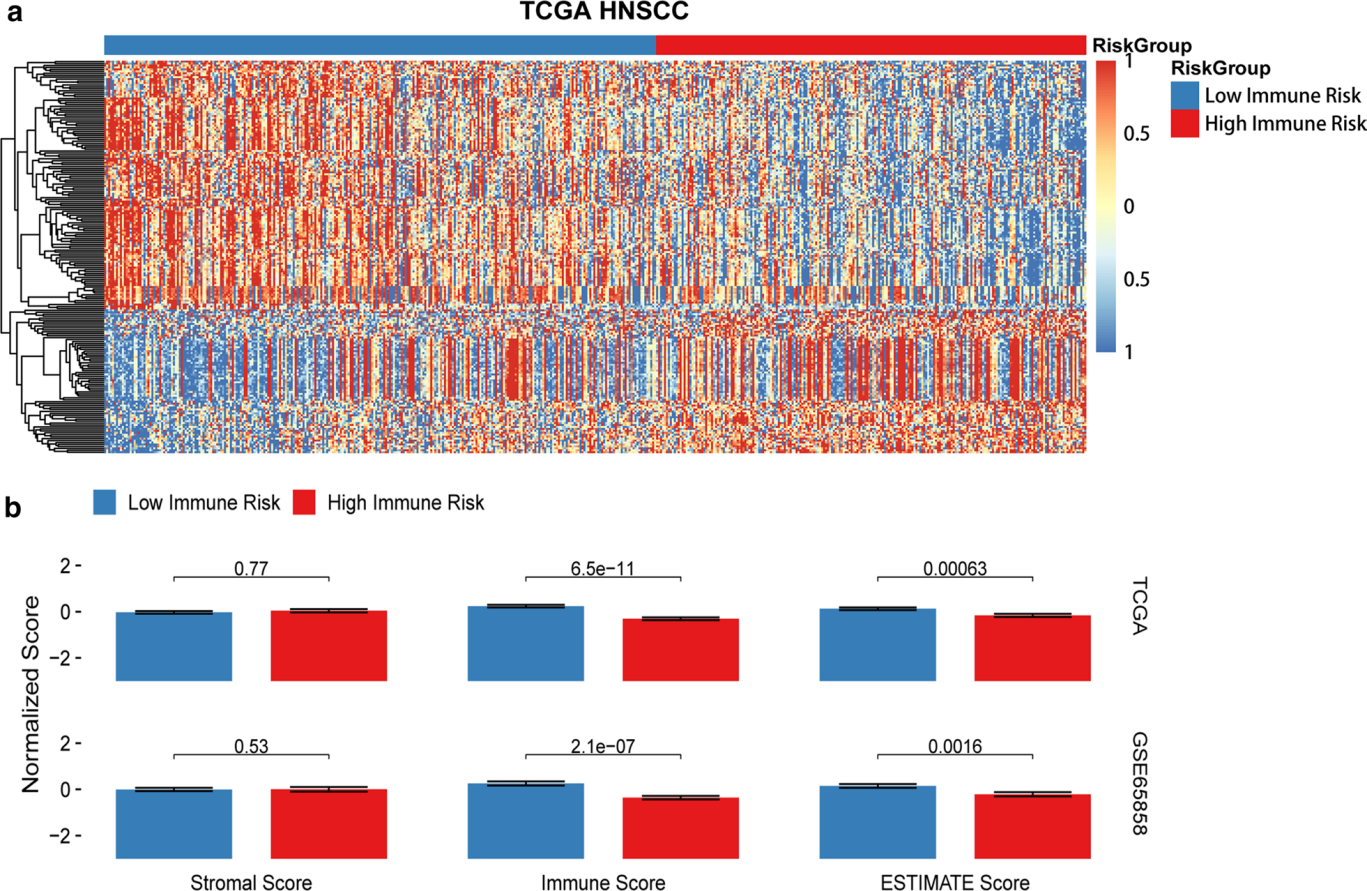
**a** Functional annotation of the IRGS. Heatmap of differentially expressed genes in two groups. **b** Analysis of ESTIMATE algorithm to the TCGA dataset

**Fig. 5:**
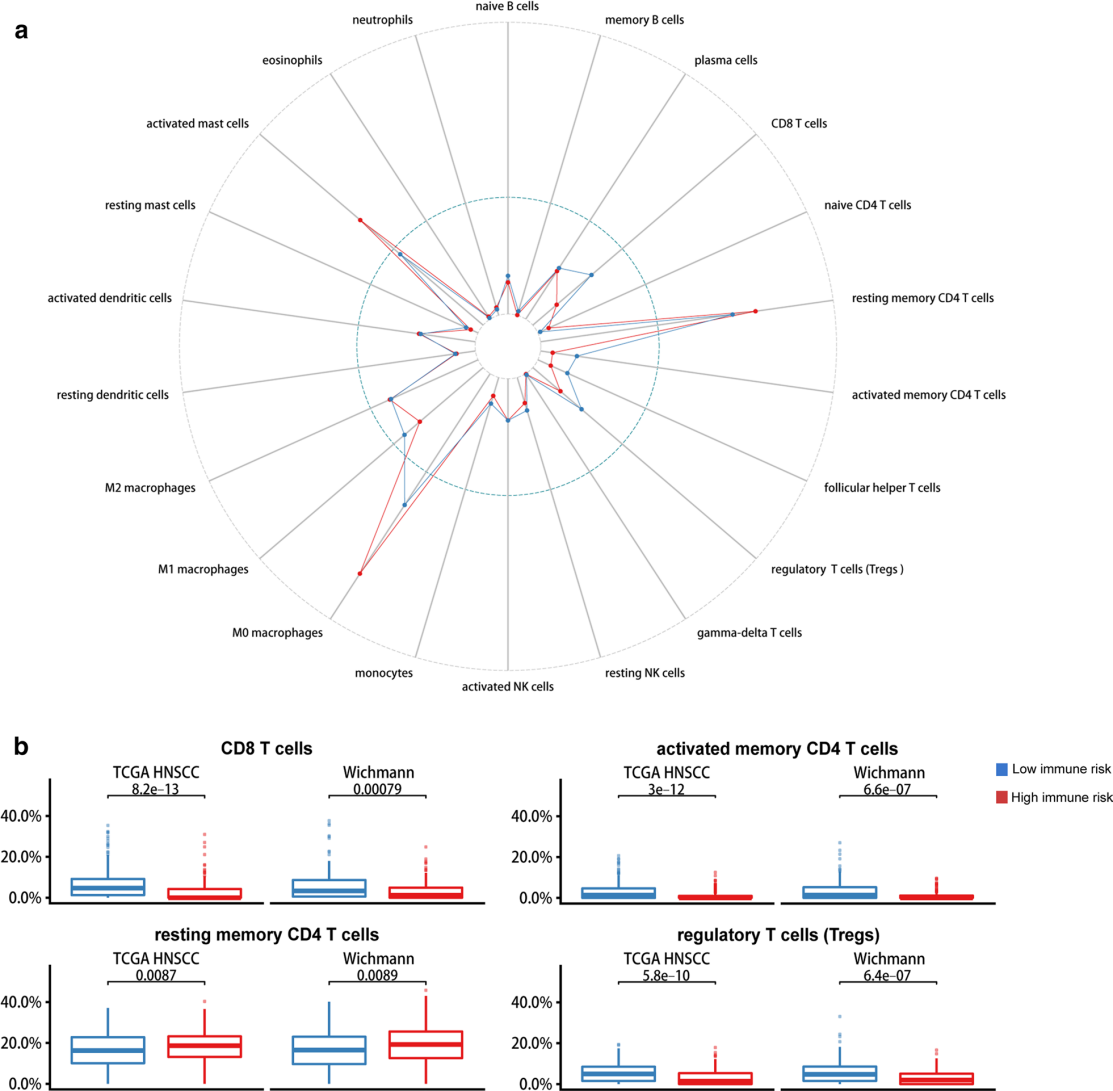
**a** Immune analysis. Immune cells are estimated based on data from TCGA. **b** The infiltration of CD8 T cells, memory-activated CD4 T cells and regulatory T cells were upregulated in the low immune group, while memory resting CD4 T cells were downregulated. *P*-values comparing immune high risk and low risk groups were calculated with t-tests

The contributions of stromal cells and immune signaling to HNSCC were estimated by the ESTIMATE algorithm. In accordance with the TCGA HNSCC data set, the IRGS showed that immune infiltration was significantly lower in the high-risk group compared to the low-risk group, with a significant difference seen for the immune score (*P *< 0.01) and no difference observed for the stromal score (*P *> 0.05) (Fig. 4b). Most notably, an immune cell type-specific analysis showed that CD8 T cells, CD4 memory activated T cells and regulatory T cells (Tregs) were highly expressed in low immune risk individuals, while CD4 memory resting T cells were enriched in the high immune risk group (*P *< 0.01, Fig. 5). In other immune-related cells, there was no statistically significant difference between the low- and high-risk groups (*P *> 0.05).

## Discussion

Reliable prognostic biomarkers are needed to identify patients with the highest risk of unfavorable survival outcomes. Numerous studies have highlighted the biomarkers associated with the pathogenesis and biology of HNSCC [14, 20–25]. Unfortunately, the accuracy of their survival evaluations remains limited and they have not yet been applied in routine clinical practice. Thus, we developed a prognostic model which incorporates 27 IRGs selected according to the ranking of gene values.

Data of HNSCC patients with different disease states and with a follow-up duration of 5 years were can be stratified into subgroups by our immune-related signature, with a high under-curve area in both the training cohort and the validation cohort. A multivariate analysis showed that incorporation of the developed immune-related signature with clinicopathological characteristics can provide a more appropriate estimation of OS in HNSCC patients. Indeed, previous findings demonstrate the improved survival HPV-positive HNSCC patients compared to patients with HPV-negative HNSCC [26]. It was found that the host immune system was influenced by remarkable downstream consequences following integration of the HPV genome into the host’s genome [26]. Specifically, an increased infiltration of immune cells and inflammatory cytokines has been recognized in the HPV-positive tumor microenvironment. This may aid better cancer clearance after irradiation [7]. Thus, HPV infection could improve the outcome of HNSCC patients. Our study, however, showed that the HPV status may be associated with the OS of HNSCC patients in the validation cohort, but not with the OS in the TCGA cohort. The information on HPV status for the TCGA cohort had been updated according to detection of viral transcripts in RNA sequencing data. One possible explanation for this may be the sample size of the TCGA and GEO datasets was very distinct. These results display that the immune signature of our study may provide a better risk prediction model compared to the HPV status.

Among these 27 genes enrolled in IRGS, six genes (ULBP1, CCR6, CCL22, ROBO1, DKK1 and PDGFA) have previously shown to correlate with the tumorigenesis of HNSCC [20–25]. As reported, CCR6 controls immune cell trafficking in reaction to inflammatory stimuli, hence determining the metastasis of HNSCC cells in vivo [21]. CCL22, an immunosuppressive cytokine, facilitates Tregs infiltration in the HPV-related tongue squamous cell carcinoma [27]. The most significant biological processes that appear to negatively correlate with the immune risk are IFN-α responses, IFN-γ responses, IL-2/STAT5 signaling and IL-6/JAK/STAT3 signaling, all of which were associated with tumor immunity. IL-6, and IFN-α/γ are prominent mediators of intercellular crosstalk [28]. IFN-γ, a key cytokine that is produced by activated T cells, natural killer (NK) cells and NK T cells, coordinates tumor immune responses [29, 30]. In the tumor microenvironment, IFN-γ signaling enhances the activation of the PD-1 signaling axis [31]. Similarly, IL6 blockade upregulates the expression of PD-L1 in melanoma cells [32]. These represent potential immunosuppressive targets to expand the therapeutic window of anti-PD-1/PD-L1 treatment. Modulation of intercellular signaling in the tumor microenvironment could be an efficacious therapeutic modality, and a simultaneous focus on these multiple therapeutic targets may mitigate the risk of a compensatory bypass in a targeted pathway [28].

Our analysis of the IRGS revealed that a higher score of immune cell infiltration was present in the low-risk group. A previous study showed that host immunosuppression is an indispensable factor of carcinogenic progression in HNSCC [32]. The microenvironment of immunodepression is characterized by the infiltration of immune cells such as Tregs [9]. Strong infiltration of forkhead/winged-helix transcription factor box P3 (FoxP3) + Tregs in HNSCC is associated with improved OS [33, 34]. Likewise, our results also demonstrate that Tregs were enriched in low immune risk groups. CD8 T cells that directly target tumor cells are robust, however, CD4 T cells in the tumor microenvironment are ambiguous for a wide range of subsets with potentially different functions [14]. Our results also indicate that CD8 T cells and memory activated CD4 T cells were highly expressed in low immune risk groups, while memory resting CD4 T cells were downregulated. Furthermore, a favorable, prognostic role of CD8 T cell infiltration was associated with better OS in HNSCC patients [14, 15, 35]. Together, our results and the results from these studies suggest that the infiltration of specific immune cells could expedite tumor progression and predict patients’ future survival rates.

As we elucidate the role of the immune system in cancer development, we can provide improved treatment strategies. In this study, we constructed a novel signature that can effectively stratify HNSCC patients into high- and low-risk groups based on clinical outcomes. It, thereby, offers a significantly improved prognostic biomarker potential compared with clinicopathologic risk factors that are currently in use. Our IRGS include stratification methods such as novel markers, specific signaling pathways and immune infiltration. Similarly, an 11-IRGs for predicting the survival of cervical cancer patients and their response to immune checkpoint inhibitors was established [36].

We would like to mention that there are some limitations in this study. First, this is a retrospective study, which is considered inferior to prospective randomized controlled clinical trials. Second, intra-tumor genetic heterogeneity supported by epigenetic and phenomenological data could lead to sampling bias. Third, despite minimization of cross-study batch effects, it needs to be noted that not all batch effects can be eliminated based on their complex nature.

## Conclusion

Taken together, our work provides a comprehensive and accurate prognosis of the immune microenvironment and survival outcomes of HNSCC patient. Our results show great promise for the identification of innovative molecular targets for immunotherapy and, hence, the improvement of treatment strategies for HNSCC patients. Further studies are needed to evaluate the clinical application of this signature for the prognosis of HNSCC.

## Supplementary information

**Additional file 1: Figure S1.** 27 immune related genes selected in LASSO COX regression.

**Additional file 2: Figure S2.** Obtaining the optimal cutoff at 5 years in a time-dependent ROC curve analysis.

**Additional file 3: Table S1.** Characteristics of patients in training and validation cohorts.

**Additional file 4: Table S2.** Univariate and multivariate analyses of IRGs, clinical and pathologic factors of patients in the validation cohort.

**Additional file 5: Table S3.** GSEA analysis showing the overexpressed biological processes in hallmarks of HNSCC.

## Data Availability

TCGA cohort data was downloaded from Broad GDAC Firehose (http://gdac.broadinstitute.org/). The datasets generated and analyzed during the current study are available in the GSE35858 (https://www.ncbi.nlm.nih.gov/geo/query/acc.cgi?acc=GSE35858).

## Abbreviations

DEGs: differentially expressed genes
FoxP3: forkhead/winged-helix transcription factor box P3
GEO: Gene Expression Omnibus
GSEA: Gene Set Enrichment Analysis
HNSCC: head and neck squamous cell carcinoma
HPV: human papillomaviruses
HR: hazard ratio
ImmPort: Immunology Database and Analysis Portal
IRGs: immune-related genes
IRGS: immune-related gene signature
LASSO: least absolute shrinkage and selection operator
OS: overall survival
ROC: receiver operating characteristic
TILs: tumor-infiltrating lymphocytes
TCGA: The Cancer Genome Atlas
Tregs: regulatory T cells
