# Supplementary figures and images for: Immune-related gene signature for predicting the prognosis of head and neck squamous cell carcinoma
Source: Cancer Cell Int. 2020 Jan 17;20:22. doi: 10.1186/s12935-020-1104-7 (PMC6969412; doi:10.1186/s12935-020-1104-7)

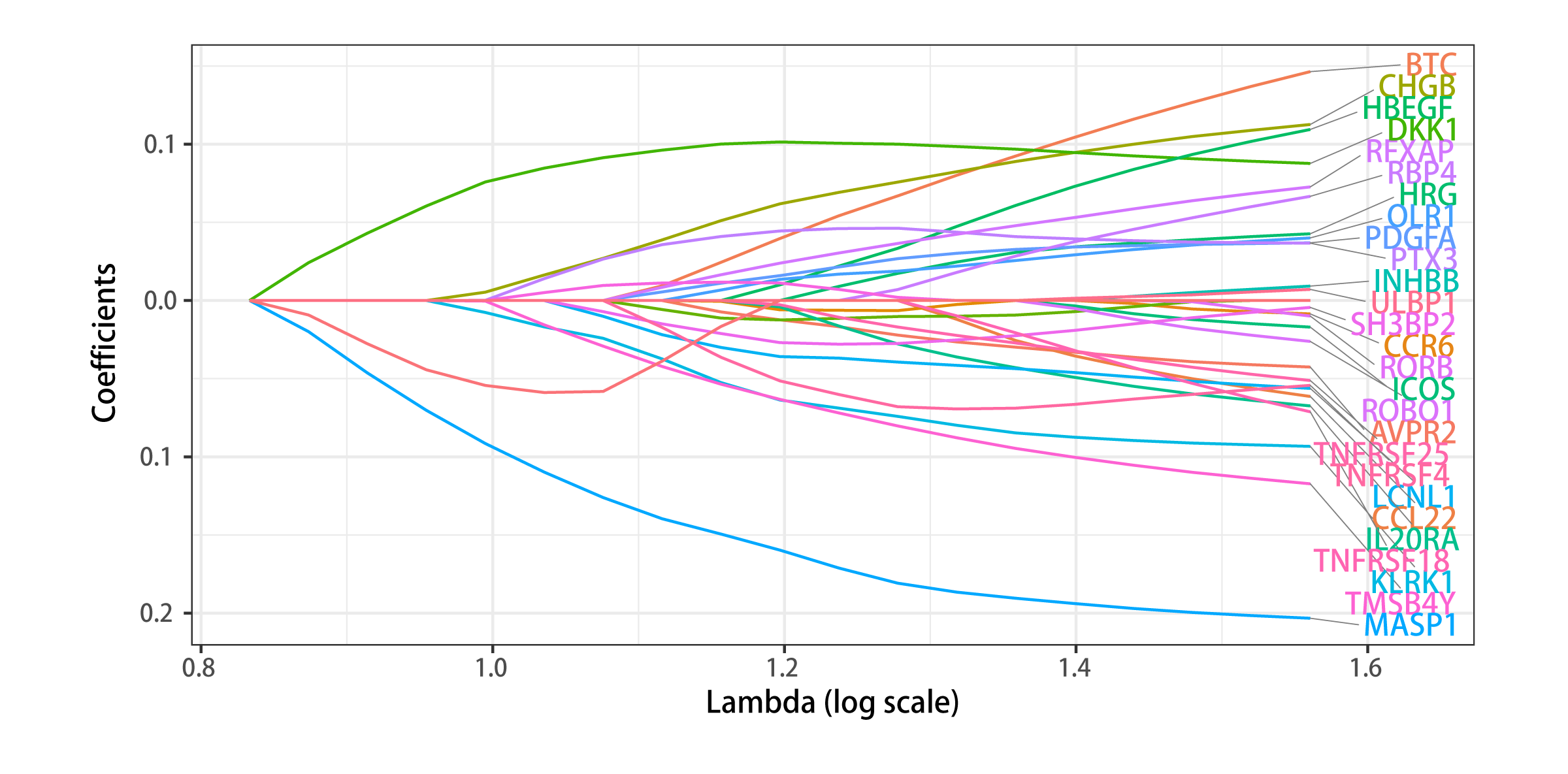

Supplement: Supplementary file 1 — Additional file 1: Figure S1. 27 immune related genes selected in LASSO COX regression. [file 12935_2020_1104_MOESM1_ESM.tif]

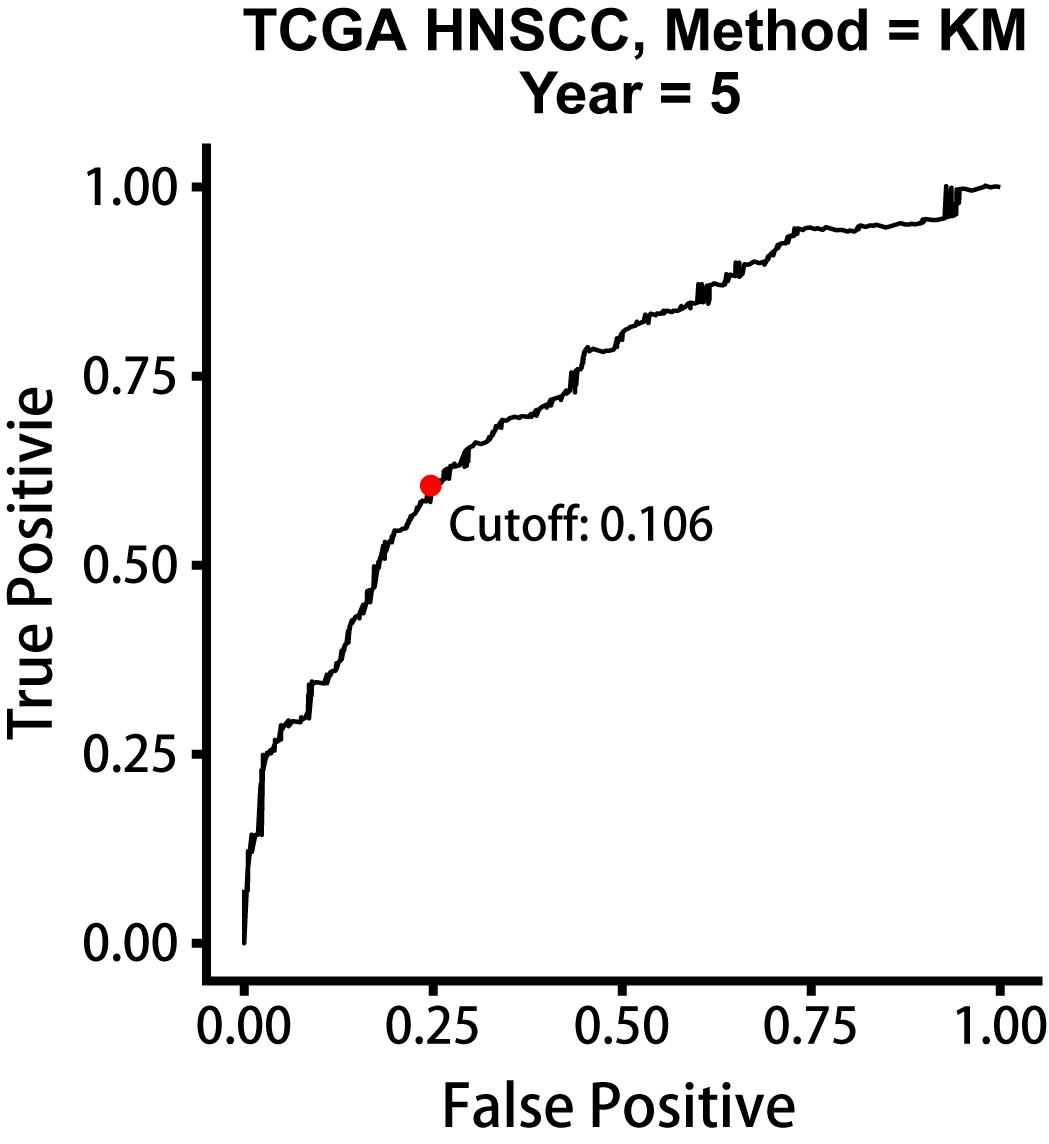

Supplement: Supplementary file 2 — Additional file 2: Figure S2. Obtaining the optimal cutoff at 5 years in a time-dependent ROC curve analysis. [file 12935_2020_1104_MOESM2_ESM.tif]
