# Supplementary material for: Immune-related gene signature for predicting the prognosis of head and neck squamous cell carcinoma
Source: Cancer Cell Int. 2020 Jan 17;20:22. doi: 10.1186/s12935-020-1104-7 (PMC6969412; doi:10.1186/s12935-020-1104-7)
Supplement: Supplementary file 3 — Additional file 3: Table S1. Characteristics of patients in training and validation cohorts. [file 12935_2020_1104_MOESM3_ESM.docx]

**Table S1.** Characteristics of patients in training and validation cohorts.

| Characteristic | TCGA | GSE65858 |  |
| --- | --- | --- | --- |
| Number of patients | 500 | 270 | |
| Patients with survival data | 499 | 270 | |
| Mean age, yrs | 61.1±11.9 | 60.1±10.3 | |
| Gender, n |  |  | |
| Male | 367 | 223 | |
| Female | 133 | 47 | |
| Tumor Location, n |  |  | |
| Alveolar Ridge | 18 | NA | |
| Base of tongue | 23 | NA | |
| Buccal Mucosa | 22 | NA | |
| Floor of mouth | 60 | NA | |
| Hard Palate | 7 | NA | |
| Hypopharynx | 10 | 33 | |
| Larynx | 111 | 48 | |
| Lip  Oral Cavity  Oral Tongue | 3  71  127 | NA  83  NA | |
| Oropharynx  Tonsil | 9  38 | 102  NA | |
| NA | 1 | 4 | |
| TNM stage, n |  |  | |
| Stage Ⅰ | 25 | 18 | |
| Stage Ⅱ | 70 | 37 | |
| Stage Ⅲ | 78 | 37 | |
| Stage Ⅳ | 259 | 178 | |
| NA | 68 | 0 | |
| Pathological grading |  |  | |
| G1 | 61 | 0 | |
| G2 | 285 | 0 | |
| G3 | 112 | 0 | |
| G4 | 2 | 0 | |
| GX | 15 | 0 | |
| NA | 25 | 270 | |
| OS event, n |  |  | |
| Yes | 499 | 270 | |
| No | 0 | 0 | |
| NA | 0 | 0 | |
| Smoking, n |  |  | |
| Yes | 443 | 222 | |
| No | 1 | 48 | |
| NA | 56 | 0 | |
| Alcohol abuse, n |  |  | |
| Yes | 310 | 240 | |
| No | 154 | 30 | |
| NA | 36 | 0 | |
| HPV status, n |  |  | |
| Negative | 319 | 196 | |
| Positive | 164 | 73 | |
| NA | 17 | 1 | |
