# Supplementary material for: Immune-related gene signature for predicting the prognosis of head and neck squamous cell carcinoma
Source: Cancer Cell Int. 2020 Jan 17;20:22. doi: 10.1186/s12935-020-1104-7 (PMC6969412; doi:10.1186/s12935-020-1104-7)
Supplement: Supplementary file 4 — Additional file 4: Table S2. Univariate and multivariate analyses of IRGs, clinical and pathologic factors of patients in the validation cohort. [file 12935_2020_1104_MOESM4_ESM.docx]

**Table S2.** Univariate and multivariate analyses of IRGs, clinicopathological factors of patients in the validation cohort.

| Characteristic | Univariate | |  | Multivariate | |
| --- | --- | --- | --- | --- | --- |
|  | HR (95%*CI*) | *P* value |  | HR (95%*CI*) | *P* value |
| IRGS | 1.84(1.21-2.81) | 0.004 |  | 1.73(1.12-2.67) | 0.014 |
| Age | 1.03(1.01-1.05) | 0.008 |  | 1.03(1.01-1.06) | 0.001 |
| Gender | 1.01(0.59-1.73) | 0.98 |  | NA | NA |
| TNM stage | 1.66(1.21-2.26) | 0.001 |  | 1.79(1.30-2.47) | <0.001 |
| Pathological grading | NA | NA |  | NA | NA |
| Smoking | 0.91(0.54-1.55) | >0.05 |  | NA | NA |
| Alcohol abuse | 1.46(0.71-3.02) | >0.05 |  | NA | NA |
| HPV | 1.95(1.15-3.33) | 0.012 |  | 2.15(1.24-3.72) | 0.006 |
