# Supplementary material for: Immune-related gene signature for predicting the prognosis of head and neck squamous cell carcinoma
Source: Cancer Cell Int. 2020 Jan 17;20:22. doi: 10.1186/s12935-020-1104-7 (PMC6969412; doi:10.1186/s12935-020-1104-7)
Supplement: Supplementary file 5 — Additional file 5: Table S3. GSEA analysis showing the overexpressed biological processes in hallmarks of HNSCC. [file 12935_2020_1104_MOESM5_ESM.docx]

Table S3. GSEA analysis showing the overexpressed biological processes in hallmarks of HNSCC.

| Gene.Set.Term | Observed.score | Pvalue | Adjusted.Pvalue |
| --- | --- | --- | --- |
| HALLMARK_EPITHELIAL_MESENCHYMAL_TRANSITION | 0.7306646 | 0 | 0 |
| HALLMARK_MYOGENESIS | 0.6739765 | 0 | 0 |
| HALLMARK_ANGIOGENESIS | 0.6485627 | 0 | 0 |
| HALLMARK_HEDGEHOG_SIGNALING | 0.5432454 | 0.006 | 0.025237 |
| HALLMARK_UV_RESPONSE_DN | 0.5414871 | 0 | 0 |
| HALLMARK_HYPOXIA | 0.5210382 | 0 | 0 |
| HALLMARK_TGF_BETA_SIGNALING | 0.4994198 | 0.006 | 0.025237 |
| HALLMARK_PROTEIN_SECRETION | 0.4901907 | 0 | 0 |
| HALLMARK_APICAL_JUNCTION | 0.4860184 | 0 | 0 |
| HALLMARK_GLYCOLYSIS | 0.4759632 | 0 | 0 |
| HALLMARK_COAGULATION | 0.4612154 | 0 | 0 |
| HALLMARK_MYC_TARGETS_V1 | 0.4342147 | 0 | 0 |
| HALLMARK_UNFOLDED_PROTEIN_RESPONSE | 0.427253 | 0 | 0 |
| HALLMARK_KRAS_SIGNALING_UP | 0.3870632 | 0 | 0 |
| HALLMARK_MTORC1_SIGNALING | 0.3864453 | 0 | 0 |
| HALLMARK_TNFA_SIGNALING_VIA_NFKB | 0.3690141 | 0.002 | 0.009795 |
| HALLMARK_MITOTIC_SPINDLE | 0.3604386 | 0.002 | 0.009795 |
